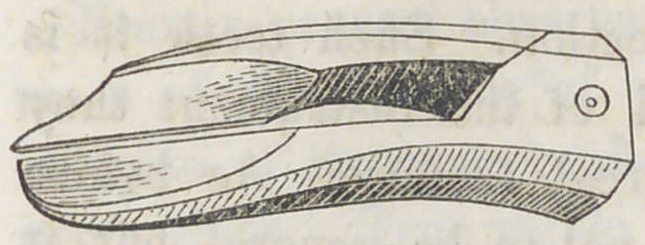# A Separating Forcep, Valuable for Extracting Dens Sapientae

**Published:** 1858-03

**Authors:** Wm. A. Pease


### A SEPARATING FORCEP VALUABLE FOR EXTRACTING DENS SAPIENTIAE.

In the August number of the Dental Recorder for 1850, is an article on "Separating Forceps for molars and bicuspids," accompanied with a cut of the forms I then used. I still continue to use them and find they are more valuable than I then supposed them to be. Subsequently I added another set to the four the beaks of which are bent in a similar manner to the forcepts designed for extracting the *Superior Dens Sapientiae*. They are all bone forceps; having sharp, cutting blades, adapted to the part and position of the tooth for which they were designed. As labor saving instruments, they are employed to separate *molars* and *bicuspids* of both *maxillars*. A person once accustomed to them will seldom use the chisel, and he will have but little need of the file, except for after-dressing the surface of the tooth. By their use he avoids unpleasant sensation and wedging of the file, the pressure of the chisel and injury to the corners of the mouth; while the separation is accomplished in a more thorough and expeditious manner. I have often thought that much of my success as a plugger of these teeth is attributable to the use of these forceps, as I was enabled to effect a complete separation of them, which gave me a strong healthy margin for the plug, and, I had, moreover, so complete access to the part next to the gum, that I was not only sure that the decay was entirely removed, but that the plug in that point was thoroughly condensed. More cases of failure arise from incomplete separation than from any other cause. The plug may be thoroughly condensed, or as a better illustration, it may be cemented, and of course, solid and fitted to the cavity; yet, the enamel frequently crumbles away down to the plug, by which it is lost, or its permanency is endangered. Complete separation is essential to good plugging—room must be had. Fancy plugging in the back part of the mouth, may be pleasant to look at as a curiosity, as a feat of manual dexterity and skill; but it is as fatal to the reputation of the dentist, as it is to the tooth. People will use their teeth improperly, and they will never understand or appreciate that skill, or control over materials, which preserves the contour of the tooth, only to have the enamel immediately crumble away and often carry with it the plug. Replugging is never profitable or reputable.

As these forceps have sharp cutting blades they are frequently useful for other purposes—splitting fangs, etc., etc.

What I now wish to make public is a new use to which I

have applied the pair, shaped like the accompaning cut. It s used to extract the *Dens Sapientiae* of both *maxillars*, in certain cases often these teeth are so much decayed as to be very difficult to extract. Caries generally commences on the luccal surface on a level with, or a little above the gum under which it often burrows, which makes the use of the ordinary forceps inapplicable or exceedingly difficult. Fracture of them is not uncommon ; after which the pain and difficulty of extracting the fangs are increased. The situation of the tooth is unfavorable for the use of the elevator in those cases where it would be indicated for the first or second *molars.* When they are much decayed their friability is so great, that there is always danger of crushing them ; and we seldom have occasion to extract them before disease has arrived at that point. It is often the case that the strongest portion of the tooth is next to, and adjoining the posterior side of the second molar. This point, when of sufficient strength, it is difficult to grasp with the ordinary forceps, as the *second molar* projects so much above it that they are carried over and beyond it. Equally difficult is it to use the elevator ; it has all that is objectionable and none of the advantages of the instrument I am describing. Perhaps the fangs of no tooth are so erratic or divergant from the usual standard, as those of the *Dens Supinetiae,* and none occasion more surprises and unlooked for complications to the extractor. Usually they have a short, stunted, tap root, describing equally to the point on all sides, and they are held in the socket as much by the attachment of the gum and periosteum as by other means; but the departures from this standard are various and frequent. When there is a crowded denture in the *inferior maxilla,* and occasionally at other times, the fangs throw out stunted spars, or rudimentary fangs in various directions, generally slender, crooked or divergent, but, the tap root often crooks backward very abruptly towards the ramus of the jaw, and, at times, when there is but one fang, it bends as abruply in the same direction. Such teeth it is difficult to extract, but with the aid of this instrument they are loosened and extracted with great facility. As before remarked, the use of this forcep is not to be general, but, it is limited to certain cases; and it is totally inapplicable unless the *second molar* remains in the mouth and it is tolerably healthy and firm in the jaw; if the *first molar* remains so much the better.

The application of the instrument is simple and easily learned. When the tooth is to be extracted, and it is decided to use it, open it and place a blade on the libial and lingual side of the tooth between the *second* and *third molars.* Let the points extend down to the gum, or if necessary, to the *alveolus*, or even so far as to grasp a slight portion of it, then bring the blades partially or entirely together, as the case indicates, which requires but a very little pressure and the tooth is loosened, then a slight prying motion throws the tooth out of the socket, when the fang is short tap shaped, or topples it over when it curves abruptly backward; after which, it can be removed with an ordinary instrument.

Comparatively little force is required to extract a difficult tooth with it, and there is much less danger of fracture than with the ordinary instruments, or elevator, because the blades are concave and press equally and evenly on both the labial and lingual side of the tooth at its strongest part near the gum next to the *second molar.* I think there is less pressure from its use on the *second molar* than from the leverage of the elevator against the adjoining tooth, and I have not observed any ill effects from it. When it is to be used exclusively for extracting, the blade should be broader than when it is designed for both extracting and separating. A pair for each purpose is best. They are valuable to me, I think they will be to others. WM. A. PEASE.

## Figures and Tables

**Figure f1:**